# An Evidence-Based Combining Classifier for Brain Signal Analysis

**DOI:** 10.1371/journal.pone.0084341

**Published:** 2014-01-02

**Authors:** Saeed Reza Kheradpisheh, Abbas Nowzari-Dalini, Reza Ebrahimpour, Mohammad Ganjtabesh

**Affiliations:** 1 Department of Computer Science, School of Mathematics, Statistics and Computer Science, University of Tehran, Tehran, Iran; 2 Brain and Intelligent Systems Research Lab, Department of Electrical and Computer Engineering, Shahid Rajaee Teacher Training University, Tehran, Iran; 3 School of Cognitive Sciences (SCS), Institute for Research in Fundamental Sciences (IPM), Tehran, Iran; University of Adelaide, Australia

## Abstract

Nowadays, brain signals are employed in various scientific and practical fields such as Medical Science, Cognitive Science, Neuroscience, and Brain Computer Interfaces. Hence, the need for robust signal analysis methods with adequate accuracy and generalizability is inevitable. The brain signal analysis is faced with complex challenges including small sample size, high dimensionality and noisy signals. Moreover, because of the non-stationarity of brain signals and the impacts of mental states on brain function, the brain signals are associated with an inherent uncertainty. In this paper, an evidence-based combining classifiers method is proposed for brain signal analysis. This method exploits the power of combining classifiers for solving complex problems and the ability of evidence theory to model as well as to reduce the existing uncertainty. The proposed method models the uncertainty in the labels of training samples in each feature space by assigning soft and crisp labels to them. Then, some classifiers are employed to approximate the belief function corresponding to each feature space. By combining the evidence raised from each classifier through the evidence theory, more confident decisions about testing samples can be made. The obtained results by the proposed method compared to some other evidence-based and fixed rule combining methods on artificial and real datasets exhibit the ability of the proposed method in dealing with complex and uncertain classification problems.

## Introduction

Different areas of the human brain are responsible for processing or controlling certain physical or mental tasks [1]. The neural activity of different brain areas is associated with the production of electrical fields around the skull. Several technologies, such as Magnetoencephalography and Electroencephalography (EEG), and Electrocorticography have been developed to measure these electrical activities. The EEG technology have been mostly welcomed by researchers because of portability, inexpensiveness, high time resolution [2].

EEG brain signals play an important role in various areas of medicine such as diagnosis and treatment of neuro-psychological disorders [3]. The EEG signals have been employed to construct Brain Computer Interfaces (BCIs) which made them popular for most of the researchers in recent years [4]. BCIs are the systems which provide a direct pathway between brain and outside devices such as computers or robotic limbs [5]. A BCI system is comprised of three essential components, signal acquisition component, signal processing component which translates brain signal into controlling commands and the external device [6].

Numerous studies [7], [8] have shown that movement and preparation for movement can block or decrease the amplitude of the ongoing mu (8–13 Hz) and beta (12–20 Hz) rhythms of EEG signal contralateral to the movement. This attenuation initiates with the movement, remains until shortly after the initiation and then returns to baseline levels within a second after the movement is started. This attenuation is called Event-Related Desynchronization (ERD) and its consecutive increase, also called Event-Related Synchronization (ERS). In addition, it is shown that ERD/ERS occurs with sensory, cognitive and other motor behaviors [7]. Therefore, the mu and beta rhythms have great potential to be used in BCI researches.

Most EEG signal applications, particularly BCI, require a signal processing system scheme to decode the brain signals recorded during mental tasks. In order to process EEG signals, like any other classification problem, several phases such as preprocessing, feature extraction, and classification are needed [9], [10]. Among these, the classification unit plays an important role in EEG signal analysis [11]. However, several issues including noisy signals, high dimensional feature space, outliers, non-stationarity of EEG, and small training samples put the brain signal classification task in trouble [12]. Moreover, uncertainty is another problem in the way of brain signal processing [13]. This uncertainty could be due to factors such as instability of mental state, lack of focus and attention, impossibility of performing a particular long term mental task and non-stationarity of brain activities.

Numerous classification algorithms with different approaches have been introduced to tackle these issues, that among them, the combining classification methods showed high potential in classifying the EEG signals [14]–[16]. Indeed, combining methods can develop a better classification system by exploiting the complementary information sources provided by base classifiers with enough diversity and accuracy. A literature review on applications of pattern recognition in EEG signal processing indicates the wide attention of researchers to use the combining methods. Numerous combining methods such as Bagging [17], Boosting [18], Random Subspace [17], Stacked Generalization [19], Majority Voting [20], [21], and Mixture of Experts [22] are applied to EEG signal classification.

There are two main strategies for combining classifiers: fusion and selection [23]. In fusion, each ensemble member is trained on the whole problem space and the final decision is made by considering the decisions of all members [23], [24]. Whereas in selection, each member is designed to learn a part of the problem space and the final decision is made by aggregating the decisions of one or some of the experts [24], [25]. Combining methods can also be categorized into two major types, hard-level and soft-level, whether the outputs of each base classifier are provided as ordered discrete class labels or as continuous values for each class, respectively [26]. Different soft-level combiners deal with the continuous outputs of base classifiers from different perspectives. Probabilistic and linear combiners interpret the classifier outputs as posteriori probabilities of each class while fuzzy [27] and evidence based [28], [29] techniques consider these values as fuzzy membership and belief values, respectively.

The Dempster-Shafer (DS) theory of evidence (also called evidence theory) is a powerful mathematical framework for dealing with uncertain information and it can be considered as a generalization of the Bayesian theory [30]. This theory can model the existing uncertainty by computing mass and belief functions instead of probability density and Bayesian probability functions. Furthermore, the DS theory allows us to reduce the level of total uncertainty by combining the evidence raised from different sources of uncertain information. After combining the several pieces of evidence, one can make decision about the class of a given sample by transforming the belief function into a probability function through Transferable Belief Model (TBM) [31].

With regards to the capabilities of DS theory to model as well as to reduce the total uncertainty using different kinds of knowledge, many researchers tend to use it in combining classifier for solving problems associated with uncertainty. Evidence-based combining classifier methods can be categorized into two groups, regarding their approach in reducing uncertainty caused by uncertain dataset or classifiers. The first category includes techniques that try to overcome the uncertainty of classifiers by computing the mass of belief pertaining to the decision of each classifier and then get a more reliable decision by combining these belief functions through the DS theory [32], [33]. For instance, Rogova [33] has approximated the mass function based on the distance between the classifier outputs for an input sample and the reference vector of each class. Each reference vector was considered as the mean of the base classifier outputs for training samples belonging to one of the main classes. The second ones are those methods trying to conquer over the inherent uncertainty of the data [29], [34]. Tabassian et. al. [29] model the uncertainty of the training samples by reassigning an imperfect label to each training sample based on the original label of its 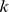-nearest samples. Afterwards, in order to simulate the corresponding belief function, a neural network was trained over the relabeled samples. By applying this procedure on different feature spaces, a set of independent sources of evidence was acquired. Combination of these complementary sources, by using DS theory, results in an efficient and generalizable classification for testing samples.

However, a limited effort in the area of brain signal analysis using evidence based classifiers have been previously performed. Yazdani et. al. [35] have used an Evidence-Based K-Nearest Neighbor (EKNN) classifier to classify EEG signals. In their method, the distance between the input pattern and each nearest training pattern is considered as an evidence that they have the same labels. Then after combining the evidences obtained by each of the nearest training patterns, the class with the highest belief value is considered as the winner class.

In this paper, we took advantage of combining classifier for solving complex problems and benefited from evidence theory in modeling and reducing the uncertainty which resulted in developing a new combining method based on evidence theory. The proposed method is used for processing the EEG signals obtained through BCI experiments and the performance is compared to some other combining methods. The obtained results of our proposed method in processing and classifying the brain signals prove its power and efficiency in solving complex problems associated with uncertainty.

The remainder of this paper is organized as follows. The Material and Methods section includes the descriptions of Dempster-Shafer theory of evidence, our proposed evidence-based combining method, and feature space selection method. Also, complete descriptions of the artificial and BCI datasets, the experiments and the settings of all methods are presented in this section. The obtained results over the artificial and real BCI datasets are provided in Results and Discussion section. Limitations of this study, future works, and the conclusions are presented in the final three sections, respectively.

## Materials and Methods

### Dempster-Shafer Theory of Evidence

Dempster-Shafer theory is a mathematical theory of evidence [36] which has shown its power in modeling the uncertainty caused by system behavior or lack of information. This theory can be considered as a generalization of probability theory where the basic probabilities are assigned to the sets of events contrary to mutually exclusive singletons. The DS theory is surrounded by several models for reasoning under uncertainty, and among them the TBM is the most applied one [31].

Let 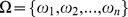 be a finite set of mutually exclusive and exhaustive hypotheses, called the frame of discernment. A mass function or Basic Belief Assignment (BBA) is a function 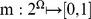 such that:
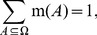
(1)where 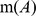 expresses the proportion of belief that is exactly assigned to 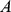 (not to any of its subsets). The set of all subsets 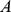 of 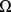 such that 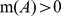 are called the focal elements of 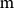. A BBA that satisfies the condition m(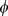) = 0, where 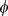 is the empty set, is called normal. Considering the BBA function, the lower and upper bounds of the probability of 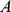 can be defined by belief and plausibility functions respectively as follows:



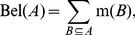
(2)

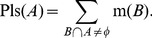
(3)


The quantity 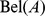 can be interpreted as total amount of probability that is allocated to 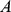, while 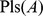 represent the maximum amount of belief that could potentially be assigned to 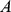. Actually, the difference between these two quantities illustrates the measure of uncertainty in determining the probability of 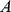.

Dempster’s rule of combination is used to combine different bodies of evidence over the same frame of discernment in order to reduce the total uncertainty. Let 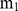 and 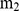 be two BBAs on 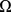, induced by two independent items of evidence. Dempster’s rule of combination (also called the orthogonal sum) that yields a new BBA 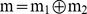 is defined as:
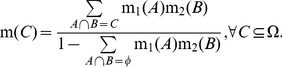
(4)


Two BBAs 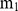 and 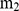 are combinable if and only if there exist at least two subsets 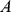 and 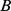 such that 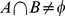, 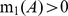, and 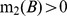.

After summarizing all pieces of evidence, a decision should be made in order to select a single hypothesis in 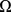. Due to existing uncertainty, one must find a transformation function that make possible to construct a probability distribution from the final belief structure [37]. For this purpose, Smets [31], [38] has introduced the pignistic transformation that is able to convince basic rational necessities. In [31], the TBM has been presented as a two-level mental model: a credal level where beliefs are represented and merged using belief functions, and a pignistic level where a decision-making process is performed. Pignistic probability is computed in the second level of the TBM. By uniformly distributing the mass of belief 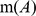 among its elements for all 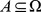, a pignistic probability distribution is defined as:
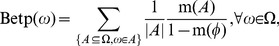
(5)where 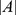 denotes the cardinality of 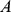 and for normal BBAs 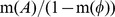 would be 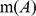 (with 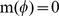).

### Proposed Method

**Figure 1:**
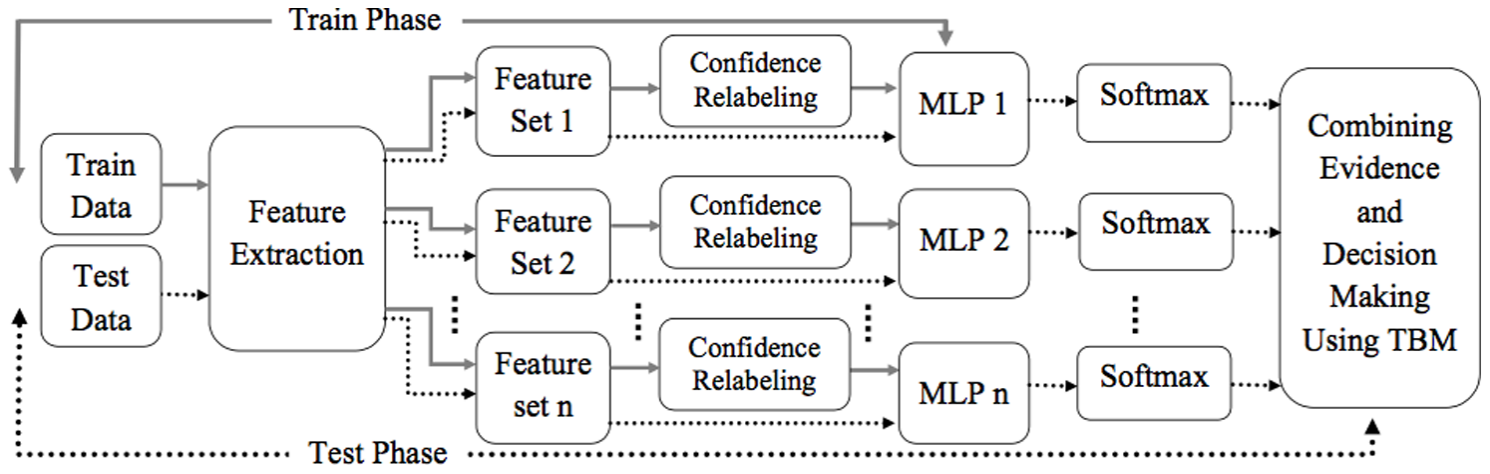
The overall scheme of the proposed method. The solid and dashed line indicate train and test phases, respectively.

In this section the proposed evidence-based classifier combination method is introduced. Figure 1 illustrates the overall scheme of the proposed method. As it can be seen in this figure, the training phase is comprised of two main modules: Confidence Relabeling and Multi Layer Perceptron (MLP) experts. In relabeling procedure, the initial labels of training samples are revised based on the level of uncertainty pertaining to the class membership of each training sample. Indeed, the level of uncertainty indicates the measure of confidence about the integrity of the labels of training samples. This procedure assigns crisp class labels to those training samples which confidently belong to a class and soft labels (any possible subset of predefined class labels) to samples with the possibility of belonging to two or more classes. Then, in order to simulate the corresponding BBA, an MLP expert is trained over the relabeled training samples. Since there are several complementary representations of the data, by performing the relabeling phase and then training an MLP classifier on each of them, a set of evidence sources with complementary information are provided. Therefore, for an input test sample in test phase, the evidence raised from several complementary representations of the data are merged through the DS theory framework, and the accepted uncertainties are reduced as results. In the rest of this section, some descriptions are given on how relabeling the training samples and training an MLP expert as a BBA function.

#### Confidence-Relabeling

Although, in the EEG signal classification problem examined in this study, each training sample has been assigned to only one class, this assignment is subject to uncertainty due to the lack of information about the mental state of the subject, the lack of control over the brain function during a specific activity, the low attention or focus of user, etc. [13], [39]. The goal of the relabeling phase is to extract the incompatibilities in the labels of training samples and model them by reassigning a crisp or soft label to each training sample based on the level of uncertainty concerning the class membership of that sample. In this subsection, the confidence-relabeling module of the proposed method is completely explained. In confidence-relabeling, first, an MLP classifier with a number of output neurons equals to the number of initial classes, is trained over the training samples. Then, the training samples are again fed into the MLP classifier, and finally, new soft or crisp labels are assigned to them based on the confidence of the MLP classifier.

Let 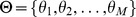 be the set of all predefined classes and 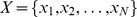 be the set of all training samples. Also assume that the subset 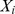 of 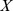 with size 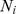 contains all training samples belonging to class 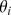 for 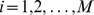. An MLP classifier is trained over training samples such that the 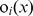, the value of 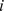-th output node, is considered as the measure of confidence concerning to class 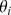 for an input data 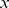. Afterwards, the template vector 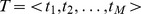 is computed where the element 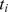 is the average value of 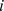-th output node over the members of 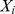, i.e.:
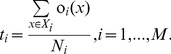
(6)


**Figure 2:**
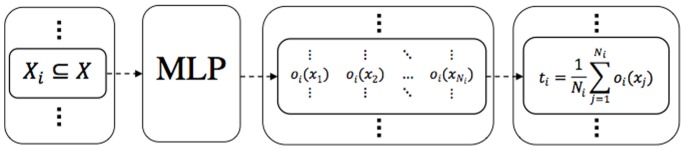
Computing the template vector 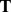. The 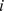-th element of the template vector 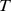 is computed by averaging the outputs of an MLP classifier over training samples of the 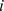-th class.

Figure 2 illustrates the process of computing the template vector 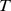. The template vector 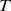 is used for computing the measure of confidence pertaining to the membership of a sample in different classes. Those training samples that are confidently classified by MLP classifier are assigned to crisp classes and those that are classified with low confidence are assigned to soft classes. Therefore, the proposed relabeling method is referred as confidence-relabeling. After computing the vector 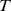, uncertainty detection and class reassignment for a training sample 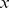 is performed as follows:

- Step 1: Apply the MLP on 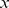 to attain the output vector 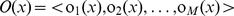, such that 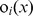 is the value of 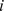-th output node, for 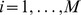.
- Step 2: Compute 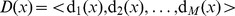 which is equal to difference between two vectors 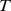 and 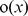, i.e.:
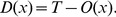
(7)
- Step 3: If the MLP classifier confidently assigns a class 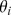 to 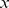, then the value of 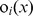 is bigger than 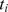. In this case, the value of element 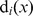 is considered as zero, in order to assign a crisp label 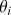 to 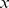 in the following steps.
- Step 4: At this step, first the minimum element of vector 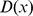 is obtained (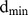) and then the value 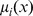 for each class is computed as follows:
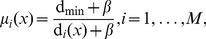
(8)where 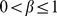 is a small constant value which helps to avoid the computational error (divide by zero) and to ensure that the value 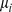 is greater than zero, even for 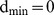 (note that 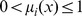). The value of 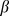 is set to 0.01 in our experiments. Also, the function 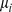 is descendant, i.e. it gets closer to one for smaller values of 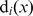.
- Step 5: Consider the threshold 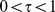 that determines the specified level of uncertainty pertaining to the training sample. If 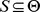 is the set of all classes, say 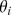, having the value of 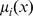 greater than 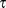, then the soft label 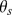 is assigned to 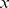, where 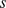 consists the indices of those classes in 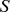. Otherwise, the situation was 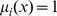 and 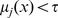 for all 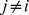, and hence the crisp label 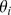 is assigned to 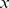.

This process must be repeated for all the training samples. Actually, small values in vector 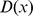 for some classes, implies an amount of uncertainty in the label of 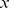. This uncertainty is modeled by assigning a soft label to this training sample. It should be noted that assigning a soft label to a subset of training samples, adds a new class to the problem space. For example, consider a classification problem with two classes in which the training sample 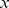 belongs to class 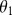 and after the relabeling phase it is reassigned to the soft class 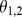. It means that the sample 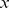 is eliminated from 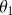 and is added to the new soft class 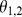.

Considering the existence of complementary information gained from different representations of the data, it is expected that if a soft label is assigned to a training sample in one feature space, this sample could belong to one class or a subset of the main classes with less uncertainty in the other feature spaces. In this case, combining the evidence raised from complementary sources of information could reduce the total uncertainty.

#### Evidence extraction and classification

In the previous section, a relabeling method was introduced to identify and model the uncertainty in labels of training samples. However, in this section, we examine how to compute and combine different sources of evidence and make decision about the labels of test samples. In training phase, after relabeling the data of each feature space, the MLP classifiers are trained over the relabeled samples to simulate the corresponding Basic Belief Assignment (BBA) functions. Indeed, for any feature space the corresponding MLP is trained over the respective training set with new crisp and soft labels. Hence, the number of output neurons of each MLP classifier is equal to the number of crisp and soft classes obtained in relabeling phase. For each sample in testing phase, each MLP can compute the measure of evidence pertaining to different classes in corresponding feature space.

Since each feature space has its own level of uncertainty, after the relabeling step, the number and type of the classes in each feature space can be different from the others. Hence, by using different feature spaces with different levels of uncertainty, independent sources of evidence could be constructed. Combining these complementary sources of evidence through the Dempster-Shafer framework reduces the accepted uncertainties and increases the classification performance.

The test phase consists of four steps. In the first step, different types of features, similar to the training phase (see Feature Space Selection subsection), are extracted from the testing sample. Second, they are applied to corresponding MLP experts to obtain measure of confidence associated with decisions about different classes. In the third step, the decision of each MLP should be converted in the form of BBA functions using softmax operator:
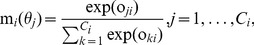
(9)where 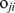 is the 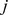-th output value of the 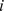-th MLP, 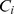 is the number of classes of the 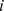-th feature space after relabeling step, and 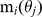 is the mass of belief given to class 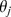 by 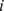-th MLP. In fact, 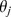 is one of the crisp or soft labels of the 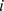-th feature space after relabeling step. In the forth step, Dempster’s rule of combination is used to merge evidence induced by all MLPs and compute the combined BBA. Then, the test sample is assigned to the predefined class with the largest pignistic probability. To compute pignistic probabilities of predefined classes, the pignistic transformation must be applied on the combined BBA.

It should be noted that the BBA function 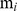, corresponding to the 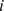-th feature space, has a focal set of elements equivalent to the obtained classes for this feature space after the relabeling step. In other words, the frame of discernment of all MLP experts is equal to the set of predefined classes, but each MLP makes decisions about some subsets of this frame which may differ from the other MLPs.

**Figure 3:**
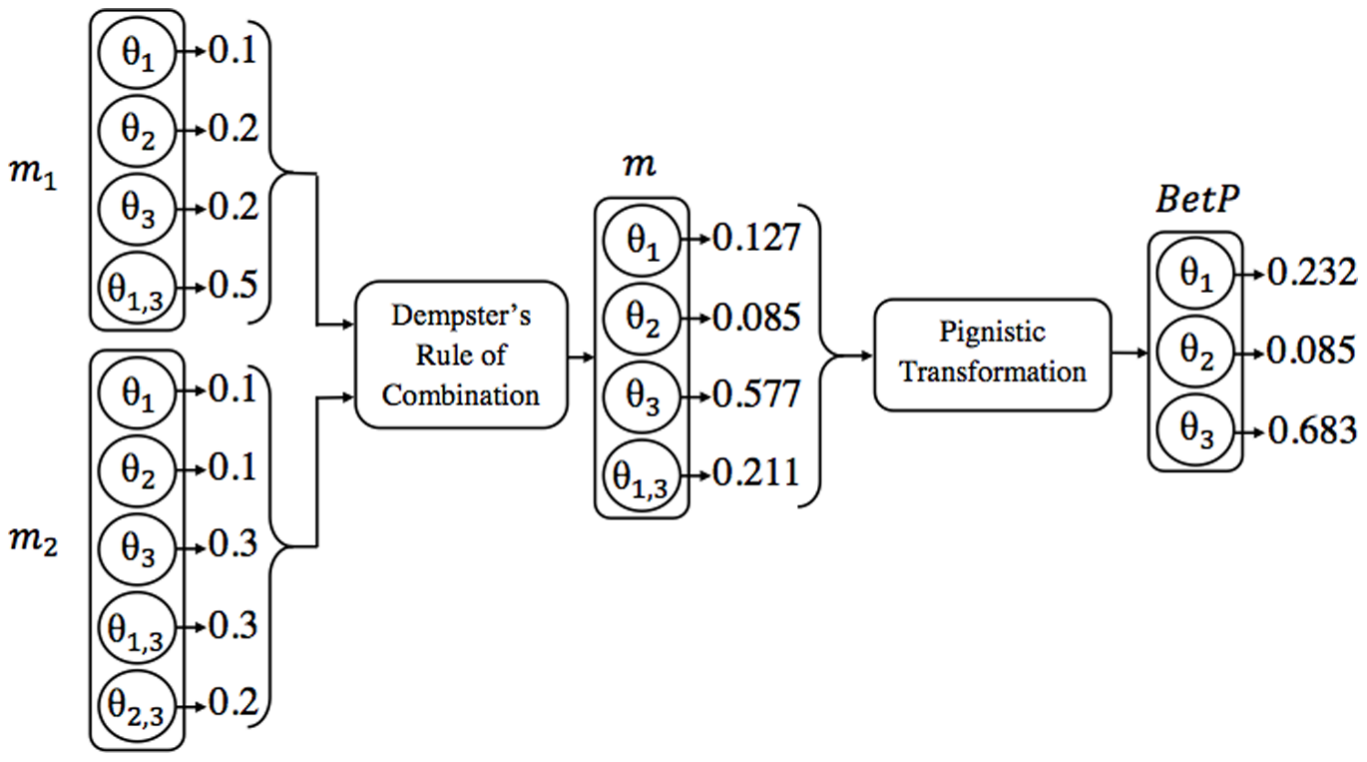
The process of making decision on a test sample for a 3-classes problem. The value of belief produced by the MLPs for each crisp or soft class as well as the final BBA obtained by merging the two bodies of evidence using Dempster’s rule of combination are presented. Decision on the test sample is made by applying pignistic transformation to the final BBA. This figure is inspired from [29].

For example consider a three-class classification problem which is presented by two different feature spaces. After the relabeling stage, two BBA functions 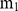 and 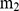 are produced by training MLP classifiers over the relabeled training samples in each feature space. The outputs of these two BBA functions for a test sample is presented in Figure 3. After combining these BBA functions through Dempster’s Rule of combination a combined BBA, 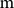, is obtained. By making use of the pignistic transformation, the value of belief corresponding to the soft class 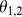 is equally distributed among its elements and finally the test sample is assigned to class 3 with the highest probability value.

### Feature Space Selection

In our proposed method, the main key to improve the performance, similar to the other combining methods, lies in the training phase of the base classifiers with an adequate trade-off between two conflicting conditions, enough accuracy and diversity [24]. Accuracy and diversity are necessary for constructing reliable and complementary sources of information, respectively. With this aim, one can select a collection of feature sets leading to diverse and efficient classifiers.

**Figure 4:**
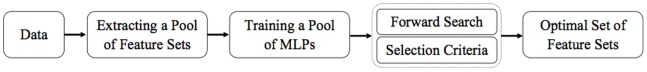
The overall scheme of the feature space selection.

Assuming that a repository of feature sets, containing different representations of the data, is available. This repository is produced by employing different feature extraction methods or by selecting different feature subsets from an original feature space. In order to select some appropriate feature sets, at first, a pool of MLP classifiers is constructed by training an MLP on each feature set within the repository. Then, in order to select the accurate classifiers, inefficient classifiers over the validation data as well as their corresponding feature sets are eliminated from the pool of classifiers and the repository of feature sets, respectively. To this end, the first 30 percent classifiers having the highest classification accuracies on the validation set are selected. Finally, by applying a search algorithm with a selection criterion (see below), an optimal set of classifiers is obtained. The corresponding feature sets of the selected classifiers are considered as the set of optimal feature sets. The overall scheme of the feature space selection is shown in Figure 4.

In this paper, a forward search algorithm with a diversity based criterion is exploited [40]. Forward search is an iterative greedy search algorithm whose remarkable performance has been examined and investigated by applying it on various experiments [41]. In the first iteration of this search algorithm, the most efficient classifier is considered as the first element of the optimal set. Then, in each iteration, one of the classifiers in the pool which has the highest diversity with the set of selected classifiers is added to this set. This process continues until the number of selected classifiers reaches to a desired number. For example, to design a system consists of five classifiers, the forward search is continued until five classifiers to be selected. To compute the diversity between classifiers, the non pairwise measure of diversity, *inter-rater agreement*, which measures the level of agreement or disagreement between classifiers is used [42]. The inter-rater agreement, 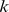, is calculated as explained below.

Let 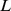 be the number of classifiers and 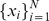 be a dataset with 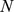 labeled samples. Assume that, 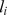 denotes the number of classifiers which correctly classify the 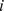-th data point, 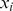. Also, let 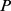 be the average classification accuracy of all classifiers. The inter-rater agreement, 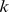, can be calculated as
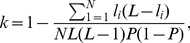
(10)where 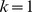 indicates dependency among classifiers and the smaller value of 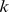 represents the better diversity among them.

### Datasets

In this subsection, the descriptions of an artificial and two real BCI datasets, which have been used in this study, are provided. The main aim of the artificial dataset is to exhibit the merits of the confidence relabeling and the proposed method. The BCI datasets are also used to illustrate the ability of the proposed method to handle the classification complexities of brain signals, specifically the uncertainty.

#### Artificial dataset

**Figure 5:**
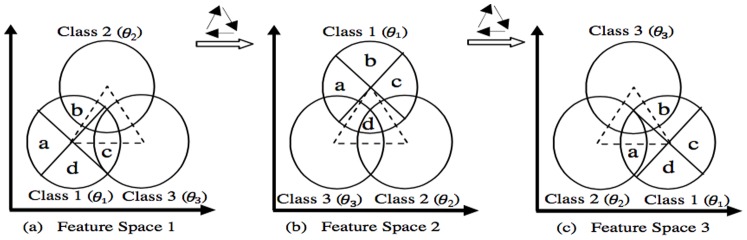
The representation of artificial dataset with three main classes, 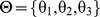. To demonstrate how different parts of a class overlap with other classes in different feature spaces, Class 1 is splited to four parts and positions of these parts in the three feature spaces are shown in a) first feature space, b) second feature space and c) third feature space. This figure is inspired from [29].

A three-class two-dimensional artificial dataset, as explained in [29], is employed to better exhibiting the relabeling procedure and its impacts on efficiency of the proposed combining method. The data contains three classes with the same sample sizes. Each class is sampled from a Gaussian distribution with identity covariance matrix, while the center of each class is placed on one of the vertices of an equilateral triangle. To generate different complementary representations of the data, two other feature spaces are made by transferring the center of each class to the next vertex in a clockwise direction, as it is shown in Figure 5. Indeed, the existing uncertainty in the labels of samples in any feature space can be reduced by other feature spaces, since a particular sample might be placed in an area with highly overlapped classes whereas, it could be placed in a less overlapping area in the other feature spaces. Moreover, to evaluate the performance of the proposed method in classification problems with different levels of uncertainty, three different dataset with high, medium, and low uncertainties are produced by varying the length of the equilateral triangle edges as 1, 2, and 3, respectively. For each level of uncertainty, 150, 300, and 500 samples are generated for train, validation and test sets, correspondingly, and then, the classes are transfered based on the aforementioned procedure to create complementary information sources.

#### Dataset V of BCI competition III

In this study, we also use the dataset V of BCI competition III, provided by IDIAP Research Institute [43]. This dataset contains EEG signal from three normal human subjects, acquired during four non-feedback sessions. The subject sat in a chair, relaxed arms resting on their legs and executed one of the following tasks:

1. Imagination of repetitive self-paced left hand movements.
2. Imagination of repetitive self-paced right hand movements.
3. Generation of words beginning with a random letter.

**Figure 6:**
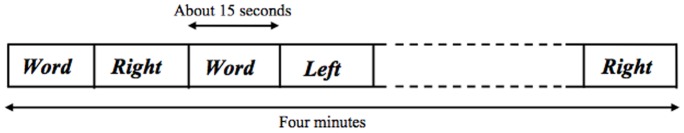
The structure of one session of the experiment.

For each subject, all four sessions were performed on the same day with 5–10 minutes breaks between sessions. Each session is comprised of a series of the described tasks and lasts about four minutes. In one session, as shown in Figure 6, the subject performed repetitively a given task for about 15 seconds and then switched randomly to another task at the operator’s request.

EEG signals were recorded using a 32-electrodes recording system, where the electrodes are located according to the international 10–20 system and the sampling rate was 512 Hz. Data are provided in two ways: (1) raw EEG signals (2) precomputed features. We use the precomputed features for our experiments in which the raw EEG signals were first spatially filtered using Surface Laplacian method to reduce large-scale scalp potentials (the aggregate signal emitted by neighboring brain areas and recorded by an electrode) and amplify localized signals. Then, every 62.5 ms, the power spectral density (PSD) [44] in the band 8–30 Hz was estimated over the last second of data with a frequency resolution of 2 Hz for the eight centroparietal channels C3, Cz, C4,CP1, CP2, P3, Pz, and P4. These features are released as the precomputed features by the dataset providers. Therefore, an EEG sample data has 96 dimensions (8 channels times 12 frequency components).

The data preparation procedure extracts 16 samples per second (every 62.5 ms with 1 second length), hence, there is one-half of a second overlap between the corresponding signals of each 8 consecutive samples. Therefore, we have down-sampled the data of all sessions by selecting the first sample of each 8 consecutive samples. In order to normalize the features, the unit length normalization technique is used in such a way that an input vector 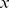 is normalized as 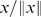. Afterward, for each subject, those samples which belong to the fourth session are considered as testing set, while the samples of earlier three sessions are used in the training phase. To train the classification system, two sets of training and validation samples are needed. Therefor, for each subject, 900 random samples of the first three sessions are extracted as training samples and the rest are considered as validation samples. Table 1 contains the class distribution of the numbers of the training, validation, and testing samples.

**Table 1:** The class distribution of the numbers of training, validation and testing samples.

Subject	Class	Training samples	Validation samples	Testing samples
1	Left	251	115	130
	Right	296	136	128
	Word	353	165	180
2	Left	261	109	108
	Right	297	129	144
	Word	342	162	182
3	Left	287	139	150
	Right	304	126	146
	Word	309	121	140

#### Dataset IIa of BCI competition IV

The Dataset IIa of BCI Competition IV [45], provided by BCI research group of Graz University, is also used in this study. The dataset consists of EEG signal recordings from nine healthy subjects, performing four different motor imagery tasks (i.e. left hand, right hand, both feet, and tongue), and obtained in two sessions on different days. Each session is comprised of six runs, and each run includes 48 trials (12 for each of the possible tasks), with a total of 288 trials per session.

The subjects were seated in an armchair in front of a computer screen. At the beginning of each trial, a fixation cross and a brief warning tone are presented to the subjects. Then, after two seconds, a cue pointing to a direction corresponding to one of the four tasks appears for 1.25 second (s). Without providing any feedback, the subject is asked to carry out the desired task until the fixation cross disappears. The EEG data was monopolarly recorded using 22 Ag/AgCl electrodes with left and right mastoids serving as reference and ground, respectively. The signals were sampled at 250 Hz and then bandpass-filtered between 0.5 Hz and 100 Hz. In addition to 22 EEG electrodes, three monopolar electro-oculogram channels were also recorded which is ignored in this study.

In order to enhance the raw EEG signal which is possibly contaminated by noise and artifacts, a surface Laplacian method is applied which is calculated by subtracting the weighted average of four surrounding channels with weights equal to the central one. Afterwards, the EEG signals were bandpass-filtered between 5 Hz and 30 Hz which covers the beta and mu rhythms in which the ERD/ERS phenomenon occurs during motor imagery tasks. To achieve this goal, a sixth order Butterworth band-pass filter is used. Signals in between 0.5 s and 2.5 s after the onset of the stimulus are considered as data samples and are used for feature extraction. Training and validation samples are drawn out from the first session, while the obtained samples from the second session are used for testing phase.

We then applied Common Spatial Pattern (CSP) [46] to extract appropriate features from the EEG signals. This method is a widely-used technique for analyzing multi-channel EEG data, specifically employed as a feature extraction method for motor imagery BCIs [47]. The original CSP is designed for two-classes problems and attempts to put emphasis on the differences between classes while suppressing the similarities between them by maximizing the variance for one class and minimizing it for the other. After computing the CSP transformation filters, the first and last 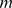 spatial filters, which lead to the highest discrimination, are used to extract the features. Three different approaches have been proposed to extend the original CSP for multiclass tasks based on one-versus-one [48], one-versus-rest [49], and approximate simultaneous diagonalization techniques [50]. One-versus-rest CSP computes 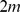 filters for each class against all others and then projects the EEG signals on all the 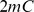 chosen filters (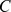 is the number of classes). In this paper, the one-versus-rest approach with 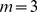 has been employed and consequently, 24 features are extracted from the EEG signal of each trial. Features of an input pattern 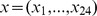 are normalized through the log-transformation as 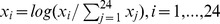. For each subject, 200 randomly selected samples of the first session is considered as training samples and the remaining samples (88 samples) are used as the validation set.

### Experimental Settings

Uncertainty of any classification system could be increased by reducing the amount of information obtained from the input data. This lack of information may be due to incompatibility of training pattern labels, extraction of inappropriate features, insufficiency of available training samples, or lack of adequate information sources. Incompatibility among the labels of training patterns naturally exists in BCI datasets due to the inherent uncertainty of the brain signals. In order to simulate the other sources of uncertainty, we have designed several experiments. It is expected that by reducing the size of the feature subsets, the amount of information carried by each training sample decreases. Therefore, we can study the impact of inappropriate features on classification systems by varying the size of the feature subsets. Also, by reducing the number of training samples, we can study the performance of the classification systems in the face of insufficient training samples. On the other hand, the number of classifiers indicates the number of information sources in a combining system, and so by varying this parameter we can investigate the relation between the information sources and the uncertainty. For each dataset, the experiments and the settings of the applied methods are presented in one of the following subsections.

#### Artificial dataset

As explained in Confidence-Relabeling subsection, the relabeling method determines the new soft or crisp labels of each training pattern based on the output of an MLP classifier trained on the whole training set. For this dataset, an MLP classifier with one hidden layer is used in confidence relabeling. The backpropagation learning rule is used to train the MLP, when the desired output values are zeros except for the actual class of the input pattern which is equal to one. The performance of the MLP on the validation set is the criteria to adjust the number of hidden neurons and the learning rate. To this end, the number of hidden neurons and learning rate are respectively varied in [3], [15] and [0.05, 0.3]. The learning procedure is stopped when the performance of the MLP over the validation set remains approximately constant or decreases for several consecutive iterations, or 1000 epochs have been completed.

Due to the availability of three complementary representations (feature spaces) of the artificial dataset in each level of uncertainty, there is no need to perform the feature space selection phase. With intention to compare the proposed method against the fixed rule combining methods such as *Maximum*, *Average* and *Product*, an MLP classifier is trained over the corresponding training samples of each feature space. Since the fixed rule methods ignore the uncertainty of training samples, the MLPs are trained over the dataset with original labels. All the MLPs have one hidden layer and are trained under backpropagation rule. The learning procedure is stopped when the performance of the MLP over the validation set remains approximately constant or decreases for several consecutive iterations, or 1000 epochs have been completed. The best value for the learning rate of the MLPs are found using a trial and error search algorithm based on the obtained performance on the validation set, when we have varied the value of this parameter in the range [0.05,0.3] with 0.05 step size. The backpropagation learning rule is also used to train the MLPs applied in relabeling phase and basic classifiers in the proposed and Tabassians’ [29] methods. The learning rate of these MLPs are also determined by a trial and error search as used for fixed rule combining methods. In addition, the parameters 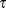 and 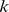 are also determined by a similar manner, where their values are varied in [0.05,0.95] and [3], [10], respectively.

#### Dataset V of BCI competition III

To study the impact of these factors, the feature subset size is changed within 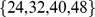 and the number of selected feature subsets is varied between 2 and 10 with the step size of one. The size of the training set is also changed within 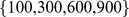, which are randomly subsampled from the whole training set.

**Figure 7:**
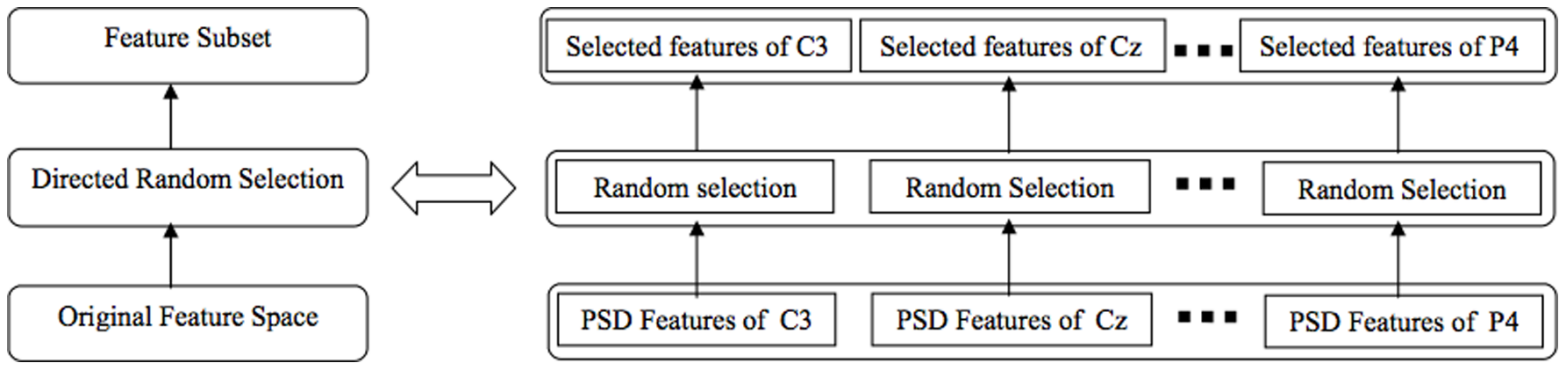
The structure of directed random feature subset generation.

For each feature subset size and each training set size, a set of optimal feature subsets should be selected via the feature space selection method. To this end, first, a repository of feature subsets with the same size are drawn from the original feature space. Each feature subset is acquired by a directed random selection procedure shown in Figure 7. Based on the directed random selection, a feature subset is generated by linking the random selected portion of PSD features of each centroparietal channel. For example, by selecting six random features from PSD vector of each channel, a 48-dimensional feature subset is obtained from the 96-dimensional original feature space. Then, a pool of MLP classifiers is generated, each of which is trained on one of the feature subsets in the repository. Finally, after elimination of inefficient MLPs from the classifier pool, the set of optimal feature subsets is selected using the forward search algorithm.

The performance of the proposed method is compared with some fixed rule combining methods such as *Average* [51], *Product* [52], *Maximum* [51], *Minimum* [51] and *Decision Templates* [53]. Further, the results of our method is compared with two evidence-based combining classifier methods proposed by Rogova [33] and Tabassian, et. al. [29]. It should be mentioned that the same feature subsets as used in our method, have been also incorporated to these combining methods.

All the MLPs used in feature space selection, relabeling, and combining methods have one hidden layer and are trained using backpropagation learning rule. For each training sample, the desired output values are zeros except for the actual class of the input pattern which is equal to one. The parameters of each MLP classifier is adjusted in a trial and error method. Actually, the number of hidden neurons and learning rate are determined based on the performance over the validation set, when their values are varied in [0.05,0.4] and [55], [56] with the step sizes of 0.05 and 1, respectively. The learning procedure is stopped when the performance of the MLP over the validation set remains approximately constant or decreases for several consecutive iterations, or 1000 epochs have been completed. In addition, the optimal value of the threshold 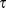 used in the relabeling stage is determined in a similar manner. The value of this threshold is changed in the range from 0.05 to 0.95 with 0.005 step size and the performance of the proposed method is evaluated for each threshold value over the validation set. The result of the best performances are considered as the final threshold settings, which are used in the test phase.

Besides, the proposed method is compared with some mostly used classification methods in BCI literature, such as Support Vector Machine (SVM) [35], Linear Discriminant Analysis (LDA) [57], MLP [23], and Evidence-based K Nearest Neighbor (EKNN) [60] classifiers. The one-against-all strategy is used for the multi-class SVM classifier. Moreover, the parameters of the SVM with Gaussian-RBF kernel, and MLP classifiers are determined using an exhaustive search based on the classification accuracy over the validation set. The regularization parameter (which adjusts the soft margin) of the SVM classifier is varied in [0.1,10] with the step size 0.1, and the variance parameter of the kernel is varied in [0.1,2] with step size 0.1. Also, for the MLP classifier, the number of hidden neurons and learning rate are varied in [10], [50] and [0.05,1] with step sizes 2 and 0.05, respectively. The value of parameter 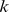 which indicates the number of considered nearest neighbor in EKNN is also selected based on the results on the validation set.

#### Dataset IIa of BCI competition IV

To study the impacts of these factors on the final performance, the feature subset size is changed in [8], [16] with the step size of 2 and the training set size is varied in 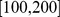 with the step size of 25. Hence, for each feature subset size and each training set size, a set of optimal feature subsets should be selected.

In order to select the set of optimal feature spaces, first of all, a repository of 100 feature subsets are randomly drawn out from the original feature space. Then, an MLP classifier is trained on each feature space to achieve a pool of classifiers. Afterward, the efficiency of each classifier over the validation set is computed and those which are not among the first 30% most efficient classifiers are eliminated from the pool. Finally, the forward search algorithm is performed on the classifier pool and those feature subsets corresponding to the selected classifiers are considered as the optimal feature subsets. The forward search algorithm continues until 5 classifiers to be selected.

In the following section, the results of the proposed method and two evidence-based combining classifier proposed by Rogova [33], and Tabassian, et. al [29] are provided. The mentioned methods are also compared with some fixed rule combining methods such as *Average* [51], *Maximum* [51], and *Decision Templates* [53]. It should be noted that, the same as proposed method, the MLP classifiers of every other combining method are trained with the optimal feature subsets which are selected by feature space selection method.

The topologies and parameters of all MLPs used in feature space selection, relabeling, and combining methods are adjusted in a similar manner as the dataset V of BCI competition III. The performance of the proposed method is also compared with some of the most applied classifiers in BCI literature such as SVM [54], LDA [55], MLP [56], and EKNN [35] classifiers. The settings of these classifiers are determined in a similar manner as dataset V of BCI competition III.

## Results and Discussion

To compare the effects of using the proposed relabeling method with respect to the previous existing relabeling method, introduced by Tabassian et. al. [29], the results of both methods over the artificial dataset is provided in Artificial Dataset subsection. Also, the performance of the proposed method along with the other methods are provided in this Subsection. In addition, to evaluate the performance of the proposed method and the other combining methods on brain EEG signals, two BCI datasets, namely dataset V of BCI competition III and dataset IIa of BCI competition IV are used. Multiple experimental circumstances are designed to appraise all the methods in different situations such as various feature set size, training sample size, and the number of base classifiers. The results over these two datasets are presented in Dataset V of BCI competition III and Dataset IIa of BCI competition IV subsections, respectively.

### Artificial Dataset

#### Crisp and soft label assignment

In this section, we would like to illustrate the function of confidence-relabeling for the artificial dataset and exhibit its advantages over KNN-relabeling proposed by Tabassian et. al. [29]. KNN-relabeling determines the new label of each training sample according to its 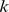 nearest training samples. Hence, for each class, the mean of those neighbors (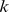-nearest training samples) belonging to the same class is considered as the local prototype of that class. Then, the similarity between the training sample and the local prototype of each class is considered as an evidence for identity of their labels. Consequently, a crisp or soft label consists of those classes whose similarity between their local prototypes and the training sample are higher than a threshold, is assigned to the training sample.

**Figure 8:**
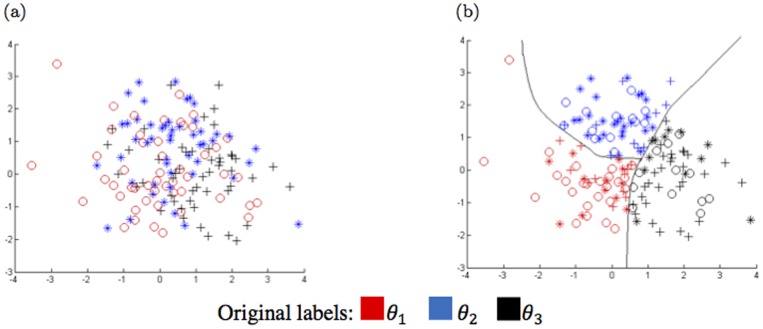
Representation of a) the training samples and b) the outputs of the MLP classifier over training set in the first feature space which has highest uncertainty.

**Figure 9:**
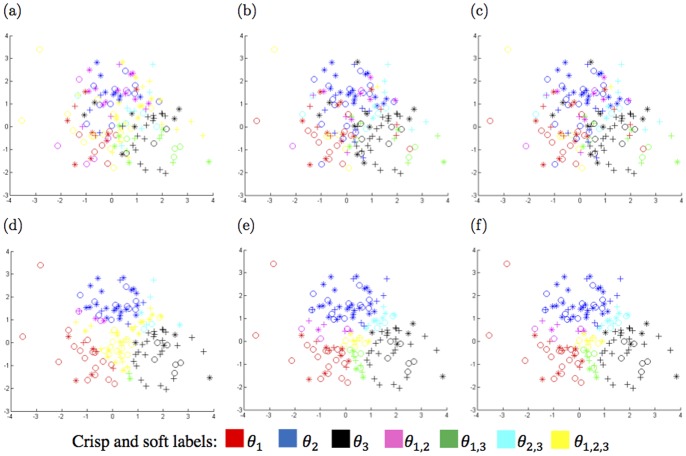
Representation of the new soft and crisp labels. Soft and crisp labels for the training samples of the first feature space of the artificial dataset with highest uncertainty computed by KNN-relabeling with a) 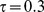, b) 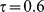 and c) 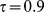, and confidence-relabeling with d) 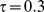, e) 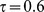 and f) 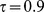.

To compare the KNN and confidence relabeling methods, their outputs for the first feature space of the artificial dataset with the highest uncertainty are considered. Figure 8a illustrates the distribution of the training samples of the main classes 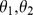, and 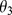 with black pluses, blue stars, and red circles, respectively. The outputs of the MLP classifier over the training samples are shown in Figure 8b where the black, blue, and red markers correspond to the training samples classified to classes 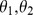, and 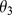, respectively. Further, to compare the behavior of confidence-relabeling against KNN-relabeling, the outputs of both methods for threshold values 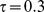, 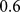 and 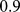 are shown in Figure 9. Both relabeling methods assigns soft or crisp labels in 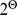 to each training samples. In these figures, the parameter 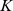 of the KNN-relabeling method is set to 5. This parameter determines the number of nearest samples participated in KNN-relabeling to generate new labels.

According to these figures, the local viewpoint of KNN-relabeling is accompanied by some drawbacks. As seen, the new soft and crisp classes determined by KNN-relabeling method are so cluttered and are not well separable. This disadvantage has negative impacts on the function of the next steps. In fact, these undesired results are caused by relabeling each training sample based on the neighboring samples which may have uncertainty in their labels. However, the global viewpoint of confidence-relabeling leads to generating separated soft and crisp classes. Typically, the confidence-relabeling explores the uncertain areas instead of finding uncertain samples. The uncertain area refers to a region of the problem space where the labels of those training samples placed in this region are contaminated by uncertainty and their soft labels, which are computed by confidence-relabeling, are the same. Uncertain samples also refer to those training samples that a soft label is assigned to them, by the relabeling module. In consequence, by considering the uncertain areas, the trained classifier over the relabeled training samples can better approximate the BBA function for the test phase.

#### Performance evaluation

**Figure 10:**
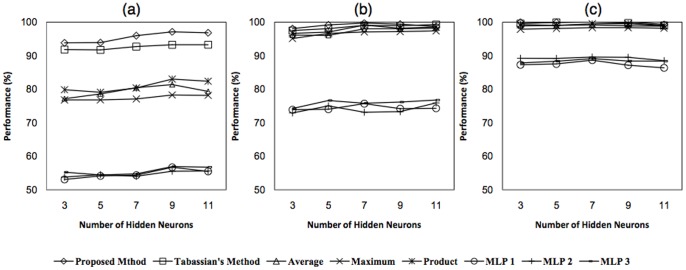
The obtained results over the artificial dataset. Classification performance as a function of the number of hidden nodes for the single MLPs trained on one of the feature spaces, fixed rule combining methods, Tabassian’s method and our proposed method. Artificial dataset with a) high level of uncertainty, b)medium level of uncertainty, c)low level of uncertainty.

**Figure 11:**
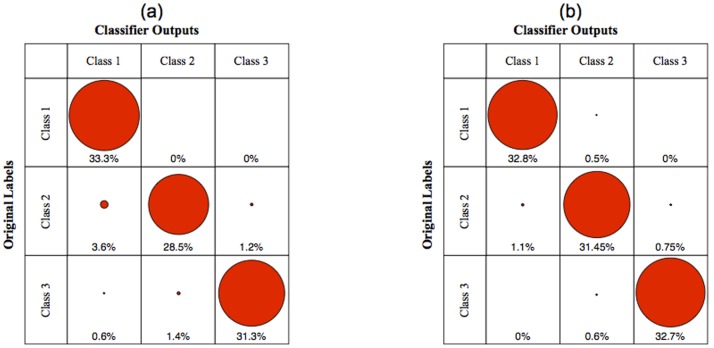
The confusion matrices of a) Tabassian’s method, and b) proposed method for the artificial dataset with the highest uncertainty.

For all three artificial datasets with high, medium, and low level of uncertainties, the performance of all fixed rules and evidence-based combining classifiers along with three single MLP classifiers trained over each feature space are illustrated in Figures 10a–10c, respectively. It is obvious that the performance of combining methods have a significant difference with respect to the performance of single MLP classifiers. This difference comes from the use of supplementary information by combining methods. It also can be seen that the evidence-based combining methods have a higher performance than fixed rule combining methods in the case of more uncertainty. The reason is due to the uncertainty reduction performed by evidence-based combining methods. By considering the presented results in Figure 10, it can be concluded that the proposed method has a better performance than evidence-based and fixed rule combining methods in classification problems concerned with uncertainty. The confusion matrices of the proposed method and Tabassian’s method in the case of high uncertainty is presented in Figure 11. The results and confusion matrices implies the superiority of the proposed method than Tabassian’s method in the presence of uncertainty. In fact, this superiority is due to the appropriate approach of the confidence-relabeling and resolving the defects of KNN-relabeling.

To assess the statistically significance of the performance improvement obtained through the proposed method with respect to the Tabassian’s method, we have synthesized the artificial dataset for 10 different times and evaluated both methods on them. Finally, we have performed a one way repeated measure ANOVA (CI = 0.95) using “method” as a factor and “performance” as the independent variable. The results indicate that the mean accuracy of the proposed method (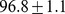) is significantly (p-value
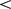
0.05) higher than the mean accuracy of the Tabassian’s method (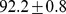), in the case of the highest uncertainty.

### Dataset V of BCI Competition III

**Figure 12:**
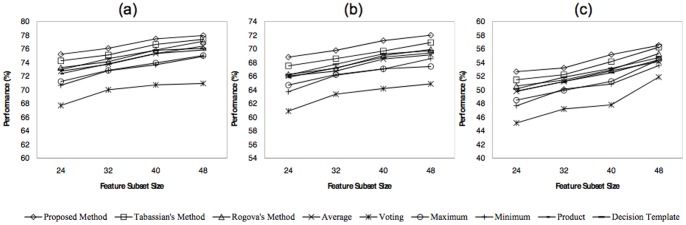
Classification performances of all methods as a function of feature subset size, for a) first b) second, and c) third subjects.

The performance of different combining methods according to different feature subset sizes are depicted in Figure 12, while the number of training samples and classifiers are fixed. Each sub-figure illustrates the performance of all methods for one of the subjects, when five base classifiers and 300 training samples are used and the size of the feature subsets are varied between 24 and 48 with the step size 8. The superiority of the proposed method in smaller feature subset sizes indicates its ability to handle the lack of information caused by inappropriate features. By increasing the size of the feature subsets, the differences between the performances of all methods decrease, which is due to the more information provided by utilizing extra features.

**Figure 13:**
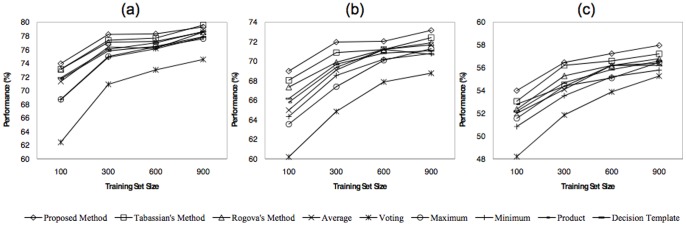
Classification performance of all methods as a function of training set size, for a) first b) second, and c) third subjects.

Figure 13 demonstrates the effects of training set size on the performance of the proposed and the other methods. The results obtained for each training set size is based on a classification system consists of five classifiers and 48-dimensional feature subset size. By considering the results in this figure, it can be concluded that the proposed method outperforms the others in the case of smaller training set size. The reason is that the uncertainty of the classification system increases by decreasing the training set size. Regarding the results presented in Figures 12 and 13, it can be concluded that the evidence-based combining methods superior the others when insufficient information is available. This lack of information may be caused by inappropriate features or inadequate training samples.

**Figure 14:**
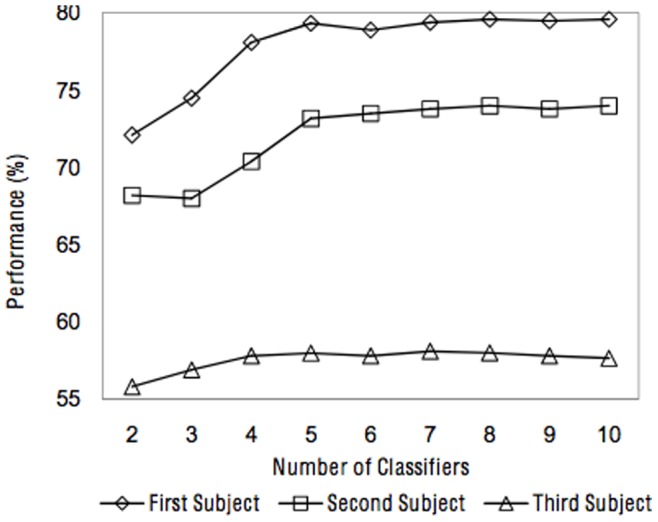
Classification performances of the proposed method according to the number applied base classifiers, for all subjects.

Each classifier in a combining system is considered as an information source which made a decision about the input pattern based on their representations in the corresponding feature space. Figure 14 provides the results of all combining methods when they exploit from 2 to 10 MLPs as their base classifiers. It should be noted that each MLP is trained over the 900 training samples represented by a different 48-dimensional feature subset which is selected using the feature subset selection method. Regarding to this figure, it can be seen that for all subjects, increasing the number of base classifiers did not necessarily lead to the performance improvement. Indeed, the amount of available information in classifiers and their diversity are more important than the number of classifiers. This figure illustrates that using about five base classifiers is enough for this dataset, if they are trained over the optimal feature subsets obtained by the feature subset selection procedure.

Now, we separately compare the above presented results of the proposed method with respect to the other evidence-based combining classifiers proposed by Rogova [33] and Tabassian et. al. [29]. All these three classification schemes make use of the same information sources and merge the decisions of their base classifiers by utilizing the Dempster’s rule of combination. Rogova’s method tries to handle the uncertainty just in the classification level and uses BBA functions which only composed of singleton classes and 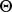, where 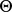 is the set of all main classes. But, our method as well as the Tabassian’s method can provide better classification performances because of using the relabeling algorithms. Actually, these two approaches are capable of considering the uncertainty associated with training samples by allowing each training sample to have a soft label including any subset of the main classes. Moreover, the proposed method outperforms the Tabassian’s method due to its global viewpoint and better operation in relabeling stage.

In order to compare the proposed method with respect to the other well-known classification methods in BCI literature such as SVM, LDA, and MLP, their performances are provided in Table 2. The proposed method is also compared with the Tabassian’s and the EKNN methods. The presented results in this table are computed based on the k-fold cross validation method. For this purpose, we have used 10-fold cross validation in such a way that, at first, the whole dataset (all four sessions) is randomly partitioned into 10 distinct folds and then the first nine folds are considered as a training set, and the last one as a test set. In order to adjust the parameters of the classification methods, one of the training folds are used as a validation set. By performing the cross validation, the performances are independent of the training set and hence, the results in Table 2 exhibit the generalization capability of all methods. The obtained results endorse that the proposed method outperforms the other methods for all subjects.

**Table 2:** The results of 10-fold cross validation for the proposed method versus Tabassian’s method, SVM, LDA, and MLP over the dataset V of BCI competition III.

Subject	Proposedmethod	Tabassian’smethod	EKNN	SVM	LDA	MLP
1	**83.1**	81.5	75.9	81.6	80.3	78.9
2	**76.9**	74.2	70.7	73.8	72.6	72.2
3	**61.5**	60.8	55.7	60.4	60.3	58.4

### Dataset IIa of BCI Competition IV

**Figure 15:**
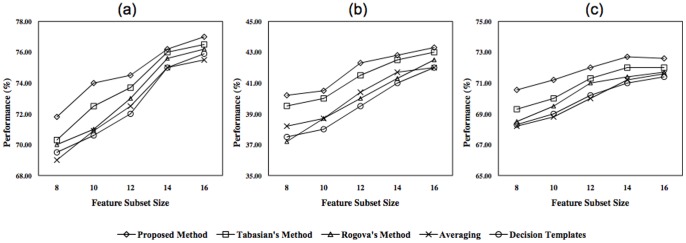
Classification performance of all methods as a function of feature subset size, for a) first b) second, and c) third subjects.

Figure 15 illustrates the performances of the proposed method along with the other combining methods as a function of feature subset size, when 100 training samples and 5 basic classifiers are used. This figure demonstrates the obtained results for the first three subjects of the desired BCI dataset. Regarding to these figures, it can be seen that by increasing the size of the feature subsets, the final performances of all methods are increased because of exploiting more informative features by the classification methods. Furthermore, it is obvious that the evidence-based methods could outperform the other methods in the case of lower feature set sizes. This fact is due to the capability of these methods in facing with problems contaminated by uncertainty which is caused by the lake of information. The superiority of the proposed method versus the Rogova’s and Tabassian’s indicates the higher capability of our method to handle the inherent uncertainty or the uncertainty imposed by insufficient information. However, the proposed and Tabassian’s methods have higher performance than the Regova’s one, due to their capability in modeling the uncertainty of the training patterns labels.

The obtained results indicate that the Tabassian’s method has the closest performance to ours. Hence, for all nine subjects, we have evaluated the proposed and Tabassian’s methods over the various feature subset sizes, and then we have performed a two way repeated measures ANOVA (CI = 0.95) using “method” (with two levels: proposed and Tabassian’s methods) and “feature subset size” (with five levels: 8, 10, 12, 14, and 16) as factors. Mauchly’s tests for the sphericity were done and the Greenhouse-Geisser correction was used if the sphericity assumption appeared to be violated. However, the results show that the difference between these two methods is significant (p-value<0.01). Also, increasing the size of the feature subsets significantly improves the performance of both methods (p-value<0.01). Regarding the superiority of the proposed method with respect to the Tabassian’s one, it can be concluded that the proposed method can significantly outperform the Tabassians’ method in the face of uncertainty caused by inappropriate features. This superiority is mainly due to the global approach of the confidence relabeling that is used by the proposed method.

**Figure 16:**
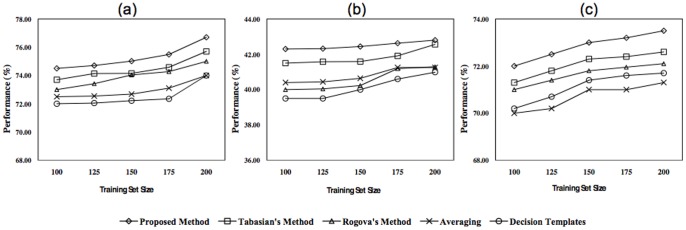
Classification performance of all methods as a function of training set size, for a) first b) second, and c) third subjects.

To evaluate the impact of the training set size on the performance of each method, the value of this parameter is changed while the size of the feature space was fixed on 12. The performance of each method as a function of the training set size is depicted in Figure 16. The results indicate that increasing the training set size leads to improve the performance of all methods, since they can exploit more information that lies in the distribution of the new training samples. In other word, using more information can reduce the measure of uncertainty in the classification system. However, based on the obtained results, the evidence-based methods have a better performance in comparison to the others, because of their capability to deal with the uncertainty of the input data and the classification system. Moreover, the proposed and the Tabassian’s methods outperform the Rogova’s method due to their ability to model and to reduce the uncertainty of input data. The Tabassian’s method has the closest performance to our method. Hence, for all nine subjects, we have evaluated the proposed and the Tabassian’s methods over various training subset sizes, and then we have performed a two way repeated measures ANOVA (CI = 0.95) using “method” (with two levels: Proposed and Tabassian’s methods) and “training subset size” (with 5 levels: 100, 125, 150, 175, and 200) as factors. Mauchly’s tests were done and if the sphericity assumption was violated then the significance test were Greenhouse-Geisser corrected. However, the results show that the achieved improvement by the proposed method is significantly (p-value
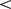
0.01) higher than the Tabassian’s method. Also, increasing the size of training set significantly (p-value
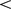
0.01) improves the final performance of both methods. Consequently, it is concluded that, although the uncertainty increases by decreasing the size of the training set, our method can significantly outperform the other methods. The superiority of the proposed method with respect to the Tabassian’s method exhibits the merits of its global view point in relabeling procedure.

The performances of the proposed method, Tabassian’s method, EKNN method [35] and some of the most applied classifiers in BCI literature such as SVM, LDA, and MLP over all nine subjects are presented in Table 3. The presented results in this table are computed based on a 10-fold cross validation method. In this way, the final performances are independent of the training set and exhibits the generalization capability of the methods. By considering these results, it can be concluded that the proposed method is superior to the other methods for all subjects.

**Table 3:** The results of 10-fold cross validation for the proposed method versus Tabassian’s method, SVM, LDA, and MLP over the dataset IIa of BCI competition IV.

Subject	Proposedmethod	Tabassian’smethod	EKNN	SVM	LDA	MLP
1	**83.2**	81.3	75.1	80.8	79.4	77.9
2	**51.9**	50.2	45.5	49.4	48.9	47.2
3	**81.8**	79.7	73.2	77.6	78.02	77.1
4	**61.3**	60.3	56.6	58.9	56.6	57.3
5	**56.7**	55.4	48.7	53.8	51.7	52.3
6	**50.2**	49.1	46.5	48.2	48.3	45.3
7	**82.5**	80.9	74.3	79.5	77.4	76.8
8	**84.2**	82.8	78.4	81.1	81.1	79.0
9	**79.3**	76.6	72.1	76.6	74.2	74.3

### Limitations of the Study

The proposed relabeling method is used to extract the uncertainty of training data. In the worst case, this method may project an 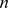-class problem into another 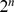-class problem. Hence, for the larger value of 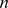, the learning phase of basic classifiers may get harder and more difficult. In addition, the computational complexity of Dempster’s rule of combination increases as the number of evidence sources increases. Therefore, the number of basic classifiers of the proposed method should be reduced in real time applications.

### Future Works

According to the power of evidence theory to handle the uncertainty, it is offered to apply the proposed method in other classification problems which suffer from the inherent uncertainty, such as biological problems. Furthermore, one can extend different parts of the proposed method and improve the possible imperfections. It is also interesting to apply evidence theory in other classification strategies such as Mixture of Experts.

## Conclusion

In this paper, an evidence-based combining method for solving classification problems associated with uncertainty has been presented. The training phase of the proposed method consists of two major parts, relabeling and MLP experts. The proposed relabeling has employed the global viewpoint of an MLP classifier to identify and model the uncertainty of the classification problem by assigning crisp and soft labels to the training samples. Then, an MLP expert is trained over training samples with new labels to simulate the BBA function. By performing the training phase on various feature spaces with complementary information, a set of independent sources of evidence is obtained. Hence, in the testing phase, the combination of evidence raised from complementary information sources thorough DS theory can reduce the total uncertainty and lead to more confident decisions about testing samples. In order to select a set of complementary sources of information, a forward search algorithm with a diversity measure as a selection criteria has been used. The performances of the proposed method on artificial and BCI datasets under different experimental conditions show the ability of the proposed method in dealing with complex and uncertain classification problems. With attention to the computational cost of the evidence-based combining methods, they are more appropriate for the off-line brain signal analysis.
